# Nitrato(1,10-phenanthroline)(1*H*-1,2,4-triazole-3-carboxyl­ato)copper(II)

**DOI:** 10.1107/S1600536808001803

**Published:** 2008-01-23

**Authors:** Jie Zhu, Xian-Hong Yin, Ya-Yan Wei, Ru-Wen Qin, Cui-Wu Lin, Hong-Feng Nong

**Affiliations:** a College of Chemistry and Ecological Engineering, Guangxi University for Nationalities, Nanning 530006, People’s Republic of China; b College of Chemistry and Chemical Engineering, Guangxi University, Nanning 530004, People’s Republic of China

## Abstract

In the title complex, [Cu(C_3_H_2_N_3_O_2_)(NO_3_)(C_12_H_8_N_2_)], the Cu^II^ ion is coordinated by an N and an O atom from a bidentate 1*H*-1,2,4-triazole-3-carboxyl­ate (TRIA) ligand, two N atoms from a 1,10-phenanthroline (phen) ligand, and an O atom from a nitrate ligand in a slightly distorted square-pyramidal environment. In the crystal structure, inter­molecular N—H⋯O hydrogen bonds link mol­ecules into one-dimensional chains propagating along the *b* axis direction.

## Related literature

For related literature, see: Guo & Wang (2005 ▶); Zhu *et al.* (2007 ▶); Zhu, Yin, Feng, Zhang *et al.* (2008 ▶); Zhu, Yin, Feng, Hu *et al.* (2008 ▶).
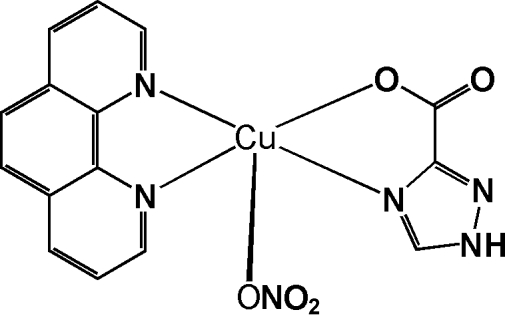


## Experimental

### 

#### Crystal data

[Cu(C_3_H_2_N_3_O_2_)(NO_3_)(C_12_H_8_N_2_)]
*M* *_r_* = 417.83
Monoclinic, 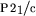

*a* = 12.3779 (14) Å
*b* = 12.6444 (15) Å
*c* = 10.0196 (10) Å
β = 107.416 (2)°
*V* = 1496.3 (3) Å^3^
*Z* = 4
Mo *K*α radiation
μ = 1.51 mm^−1^
*T* = 298 (2) K
0.34 × 0.30 × 0.25 mm

#### Data collection

Bruker SMART CCD diffractometer
Absorption correction: multi-scan (*SADABS*; Sheldrick, 1996 ▶) *T*_min_ = 0.628, *T*_max_ = 0.704
7489 measured reflections
2601 independent reflections
2102 reflections with *I* > 2σ(*I*)
*R*_int_ = 0.033

#### Refinement

*R*[*F* ^2^ > 2σ(*F* ^2^)] = 0.029
*wR*(*F* ^2^) = 0.086
*S* = 1.06
2601 reflections
244 parameters
H-atom parameters constrained
Δρ_max_ = 0.31 e Å^−3^
Δρ_min_ = −0.29 e Å^−3^

### 

Data collection: *SMART* (Siemens, 1996 ▶); cell refinement: *SAINT* (Siemens, 1996 ▶); data reduction: *SAINT*; program(s) used to solve structure: *SHELXS97* (Sheldrick, 2008 ▶); program(s) used to refine structure: *SHELXL97* (Sheldrick, 2008 ▶); molecular graphics: *PLATON* (Spek, 2003 ▶); software used to prepare material for publication: *SHELXTL* (Sheldrick, 2008 ▶).

## Supplementary Material

Crystal structure: contains datablocks I, global. DOI: 10.1107/S1600536808001803/lh2590sup1.cif

Structure factors: contains datablocks I. DOI: 10.1107/S1600536808001803/lh2590Isup2.hkl

Additional supplementary materials:  crystallographic information; 3D view; checkCIF report

## Figures and Tables

**Table 1:** Selected bond lengths (Å)

Cu1—O1	1.9540 (19)
Cu1—N4	1.988 (2)
Cu1—N3	2.005 (2)
Cu1—N5	2.015 (2)
Cu1—O3	2.315 (2)

**Table 2:** Hydrogen-bond geometry (Å, °)

*D*—H⋯*A*	*D*—H	H⋯*A*	*D*⋯*A*	*D*—H⋯*A*
N1—H1⋯O2^i^	0.86	1.92	2.775 (3)	172

Symmetry code: (i) 
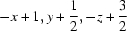
.

**Fig. 1.**
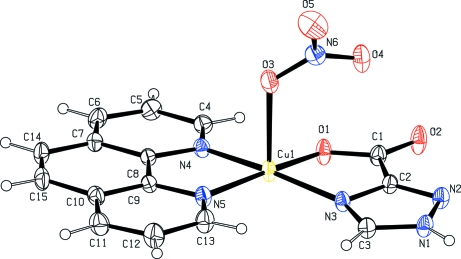
The molecular structure of the title compound showing 30% probability displacement ellipsoids and the atom-numbering scheme.

**Fig. 2.**
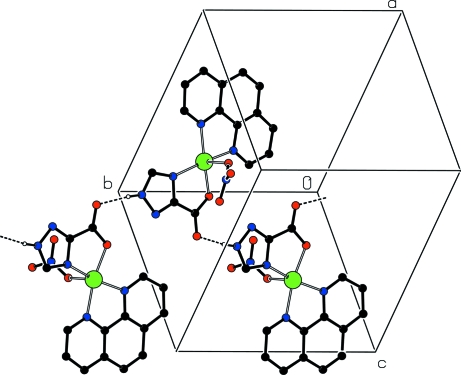
Part of the crystal structure of the title compound showing hydrogen bonds as dashed lines.

## supplementary crystallographic information

### Comment

In connection with our on-going studies in coordination chemistry (Zhu *et al.*, 2007; Zhu, Yin, Feng, Hu *et al.*, 2008; Zhu, Yin, Feng, Zhang *et al.*, 2008) and the biological importance of triazole molecules (Guo *et al.*, 2005), the crystal structure of a new ternary Cu(II) complex with 1*H*-1,2,4-triazole-3-carboxylate (TRIA), 1,10-phenanthroline (phen) and NO_3_ ligands is described. The molecular structure of the title compound is shown in Fig. 1. The Cu^II^ ion is bis-chelated by an N and an O atom, from a TRIA ligand, two N atoms from the chelating phen ligand, and the coordination geometry is completed by a O atom from an NO_3_ ligand. The atom O3 from the NO_3_ ligand occupies the apical site in a slightly distorted square-pyramidal ON_3_O coordination environment. The primary intermolecular contacts in the crystal structure are of the type N—H···O and involve the non-coordinating O atom of the carbonyl group and the N—H group of the TRIA ligand.

### Experimental

CuNO_3_.3H_2_O (0.5 mmol, 120.8 mg) dissolved in distilled water (5 ml) was added with stirring at 323 K to 1*H*-1,2,4-triazole-3-carboxylic acid (0.5 mmol, 56.5 mg) also dissolved in distilled water (15 ml). The resulting blue solution was allowed to react for 30 min and 1,10-phenanthroline (0.5 mmol, 99.1 mg) dissolved in ethanol (5 ml) was added. Dark-blue crystals suitable for X-ray analysis were obtained by slow evaporation over a period of one month (yield 55%). Analysis. Found: C 43.28, H 2.22, N 20.33, O 19.01%. C_15_H_10_CuN_6_O_5_ requires: C 43.12, H 2.41, N 20.11, O 19.15%.

### Refinement

H atoms were placed in calculated positions and included in the refinement in the riding-model approximation with N–H = 0.86 Å and C—H = 0.93 Å, and with *U*_iso_(H) 1.2*U*_eq_(C,*N*).

### Crystal data

**Table:**

[Cu(C_3_H_2_N_3_O_2_)(NO_3_)(C_12_H_8_N_2_)]	*F*_000_ = 844
*M**_r_* = 417.83	*D*_x_ = 1.855 Mg m^−^^3^
Monoclinic, *P*2_1_/*c*	Mo *K*α radiation λ = 0.71073 Å
Hall symbol: -P 2ybc	Cell parameters from 3888 reflections
*a* = 12.3779 (14) Å	θ = 2.7–27.7º
*b* = 12.6444 (15) Å	µ = 1.51 mm^−^^1^
*c* = 10.0196 (10) Å	*T* = 298 (2) K
β = 107.416 (2)º	Block, dark-blue
*V* = 1496.3 (3) Å^3^	0.34 × 0.30 × 0.25 mm
*Z* = 4	

### Data collection

**Table:**

Bruker SMART CCD diffractometer	2601 independent reflections
Radiation source: fine-focus sealed tube	2102 reflections with *I* > 2σ(*I*)
Monochromator: graphite	*R*_int_ = 0.033
*T* = 298(2) K	θ_max_ = 25.0º
φ and ω scans	θ_min_ = 1.7º
Absorption correction: multi-scan(SADABS; Sheldrick, 1996)	*h* = −14→8
*T*_min_ = 0.628, *T*_max_ = 0.704	*k* = −14→15
7489 measured reflections	*l* = −11→11

### Refinement

**Table:**

Refinement on *F*^2^	Secondary atom site location: difference Fourier map
Least-squares matrix: full	Hydrogen site location: inferred from neighbouring sites
*R*[*F*^2^ > 2σ(*F*^2^)] = 0.030	H-atom parameters constrained
*wR*(*F*^2^) = 0.086	*w* = 1/[σ^2^(*F*_o_^2^) + (0.0436*P*)^2^ + 0.8762*P*] where *P* = (*F*_o_^2^ + 2*F*_c_^2^)/3
*S* = 1.06	(Δ/σ)_max_ = 0.001
2601 reflections	Δρ_max_ = 0.31 e Å^−^^3^
244 parameters	Δρ_min_ = −0.29 e Å^−^^3^
Primary atom site location: structure-invariant direct methods	Extinction correction: none

### Special details

**Table:**

Geometry. All e.s.d.'s (except the e.s.d. in the dihedral angle between two l.s. planes) are estimated using the full covariance matrix. The cell e.s.d.'s are taken into account individually in the estimation of e.s.d.'s in distances, angles and torsion angles; correlations between e.s.d.'s in cell parameters are only used when they are defined by crystal symmetry. An approximate (isotropic) treatment of cell e.s.d.'s is used for estimating e.s.d.'s involving l.s. planes.
Refinement. Refinement of *F*^2^ against ALL reflections. The weighted *R*-factor *wR* and goodness of fit *S* are based on *F*^2^, conventional *R*-factors *R* are based on *F*, with *F* set to zero for negative *F*^2^. The threshold expression of *F*^2^ > σ(*F*^2^) is used only for calculating *R*-factors(gt) *etc*. and is not relevant to the choice of reflections for refinement. *R*-factors based on *F*^2^ are statistically about twice as large as those based on *F*, and *R*- factors based on ALL data will be even larger.

### Fractional atomic coordinates and isotropic or equivalent isotropic displacement parameters (Å^2^)

**Table:**

	*x*	*y*	*z*	*U*_iso_*/*U*_eq_	
Cu1	0.67882 (3)	1.02713 (2)	0.58353 (3)	0.02944 (14)	
N1	0.57639 (19)	1.32426 (17)	0.6760 (2)	0.0321 (5)	
H1	0.5704	1.3918	0.6815	0.039*	
N2	0.52709 (19)	1.25284 (17)	0.7412 (2)	0.0309 (5)	
N3	0.62666 (18)	1.17198 (17)	0.6177 (2)	0.0279 (5)	
N4	0.69970 (18)	0.87706 (17)	0.5374 (2)	0.0277 (5)	
N5	0.78098 (19)	1.05325 (17)	0.4636 (2)	0.0298 (5)	
N6	0.8485 (2)	1.07924 (19)	0.8885 (2)	0.0360 (6)	
O1	0.56644 (17)	0.98205 (14)	0.6733 (2)	0.0369 (5)	
O2	0.46508 (19)	1.03880 (15)	0.8074 (2)	0.0449 (6)	
O3	0.83942 (18)	1.03049 (17)	0.7753 (2)	0.0451 (5)	
O4	0.76459 (19)	1.08825 (19)	0.9301 (2)	0.0512 (6)	
O5	0.9405 (2)	1.1152 (2)	0.9554 (3)	0.0639 (7)	
C1	0.5273 (2)	1.0534 (2)	0.7344 (3)	0.0304 (6)	
C2	0.5598 (2)	1.1626 (2)	0.7031 (3)	0.0268 (6)	
C3	0.6344 (2)	1.2759 (2)	0.6036 (3)	0.0315 (6)	
H3	0.6746	1.3093	0.5507	0.038*	
C4	0.6555 (2)	0.7905 (2)	0.5753 (3)	0.0312 (6)	
H4A	0.6063	0.7972	0.6290	0.037*	
C5	0.6811 (2)	0.6898 (2)	0.5364 (3)	0.0355 (7)	
H5A	0.6479	0.6306	0.5629	0.043*	
C6	0.7544 (2)	0.6778 (2)	0.4597 (3)	0.0367 (7)	
H6	0.7714	0.6107	0.4336	0.044*	
C7	0.8041 (2)	0.7676 (2)	0.4206 (3)	0.0300 (6)	
C8	0.7725 (2)	0.8658 (2)	0.4602 (3)	0.0255 (6)	
C9	0.8155 (2)	0.9609 (2)	0.4195 (2)	0.0261 (6)	
C10	0.8887 (2)	0.9563 (2)	0.3374 (3)	0.0314 (6)	
C11	0.9255 (3)	1.0523 (3)	0.2966 (3)	0.0403 (7)	
H11	0.9718	1.0534	0.2387	0.048*	
C12	0.8919 (3)	1.1448 (2)	0.3435 (3)	0.0427 (8)	
H12	0.9169	1.2092	0.3190	0.051*	
C13	0.8209 (2)	1.1429 (2)	0.4275 (3)	0.0375 (7)	
H13	0.8005	1.2066	0.4597	0.045*	
C14	0.8817 (2)	0.7653 (2)	0.3406 (3)	0.0371 (7)	
H14	0.9051	0.7004	0.3153	0.044*	
C15	0.9220 (2)	0.8549 (2)	0.3007 (3)	0.0368 (7)	
H15	0.9725	0.8508	0.2483	0.044*	

### Atomic displacement parameters (Å^2^)

**Table:**

	*U*^11^	*U*^22^	*U*^33^	*U*^12^	*U*^13^	*U*^23^
Cu1	0.0405 (2)	0.01967 (19)	0.0373 (2)	−0.00062 (14)	0.02555 (16)	−0.00268 (14)
N1	0.0420 (14)	0.0163 (11)	0.0440 (13)	0.0013 (10)	0.0220 (12)	−0.0007 (10)
N2	0.0386 (13)	0.0201 (11)	0.0407 (13)	0.0001 (10)	0.0221 (11)	0.0015 (10)
N3	0.0353 (13)	0.0223 (11)	0.0327 (12)	−0.0008 (9)	0.0202 (10)	−0.0008 (9)
N4	0.0314 (12)	0.0261 (12)	0.0292 (11)	−0.0020 (10)	0.0146 (10)	−0.0007 (10)
N5	0.0370 (13)	0.0252 (12)	0.0329 (12)	−0.0017 (10)	0.0190 (11)	−0.0019 (10)
N6	0.0483 (16)	0.0258 (12)	0.0368 (14)	−0.0026 (11)	0.0172 (13)	0.0039 (11)
O1	0.0511 (13)	0.0207 (10)	0.0525 (12)	−0.0022 (8)	0.0361 (11)	−0.0036 (9)
O2	0.0627 (15)	0.0243 (11)	0.0683 (14)	0.0007 (9)	0.0509 (12)	0.0013 (10)
O3	0.0444 (13)	0.0571 (15)	0.0368 (11)	0.0069 (10)	0.0165 (10)	−0.0106 (10)
O4	0.0593 (15)	0.0496 (14)	0.0559 (14)	0.0053 (11)	0.0343 (12)	−0.0101 (11)
O5	0.0640 (17)	0.0648 (17)	0.0596 (15)	−0.0248 (14)	0.0132 (13)	−0.0089 (13)
C1	0.0362 (16)	0.0225 (13)	0.0379 (15)	0.0015 (11)	0.0194 (13)	0.0012 (12)
C2	0.0298 (14)	0.0226 (14)	0.0310 (14)	0.0013 (11)	0.0140 (12)	−0.0009 (11)
C3	0.0387 (16)	0.0241 (14)	0.0367 (15)	−0.0015 (12)	0.0189 (13)	0.0011 (12)
C4	0.0370 (16)	0.0276 (14)	0.0306 (14)	−0.0006 (12)	0.0129 (13)	0.0009 (11)
C5	0.0420 (17)	0.0244 (14)	0.0406 (16)	−0.0032 (12)	0.0134 (14)	0.0017 (12)
C6	0.0425 (18)	0.0239 (14)	0.0427 (16)	0.0052 (12)	0.0109 (14)	−0.0039 (12)
C7	0.0340 (15)	0.0281 (15)	0.0292 (14)	0.0029 (12)	0.0112 (12)	−0.0035 (11)
C8	0.0267 (14)	0.0271 (14)	0.0237 (13)	0.0011 (11)	0.0090 (11)	−0.0028 (11)
C9	0.0290 (14)	0.0270 (14)	0.0243 (13)	0.0001 (11)	0.0109 (11)	−0.0019 (11)
C10	0.0284 (15)	0.0396 (17)	0.0291 (14)	−0.0008 (12)	0.0130 (12)	−0.0023 (12)
C11	0.0413 (18)	0.0448 (19)	0.0421 (17)	−0.0041 (14)	0.0237 (15)	0.0021 (14)
C12	0.0499 (19)	0.0366 (17)	0.0524 (19)	−0.0053 (14)	0.0319 (16)	0.0055 (14)
C13	0.0464 (18)	0.0246 (15)	0.0483 (18)	−0.0022 (12)	0.0245 (15)	−0.0018 (13)
C14	0.0417 (17)	0.0338 (16)	0.0390 (16)	0.0071 (13)	0.0169 (14)	−0.0092 (13)
C15	0.0340 (16)	0.0440 (18)	0.0389 (16)	0.0040 (13)	0.0207 (13)	−0.0072 (13)

### Geometric parameters (Å, °)

**Table:**

Cu1—O1	1.9540 (19)	C3—H3	0.9300
Cu1—N4	1.988 (2)	C4—C5	1.396 (4)
Cu1—N3	2.005 (2)	C4—H4A	0.9300
Cu1—N5	2.015 (2)	C5—C6	1.362 (4)
Cu1—O3	2.315 (2)	C5—H5A	0.9300
N1—C3	1.314 (3)	C6—C7	1.402 (4)
N1—N2	1.361 (3)	C6—H6	0.9300
N1—H1	0.8600	C7—C8	1.395 (4)
N2—C2	1.305 (3)	C7—C14	1.424 (4)
N3—C3	1.329 (3)	C8—C9	1.423 (4)
N3—C2	1.363 (3)	C9—C10	1.396 (4)
N4—C4	1.329 (3)	C10—C11	1.401 (4)
N4—C8	1.359 (3)	C10—C15	1.427 (4)
N5—C13	1.329 (4)	C11—C12	1.371 (4)
N5—C9	1.362 (3)	C11—H11	0.9300
N6—O5	1.223 (3)	C12—C13	1.387 (4)
N6—O4	1.234 (3)	C12—H12	0.9300
N6—O3	1.267 (3)	C13—H13	0.9300
O1—C1	1.266 (3)	C14—C15	1.347 (4)
O2—C1	1.225 (3)	C14—H14	0.9300
C1—C2	1.497 (4)	C15—H15	0.9300
			
O1—Cu1—N4	89.34 (8)	N4—C4—C5	121.7 (3)
O1—Cu1—N3	82.99 (8)	N4—C4—H4A	119.2
N4—Cu1—N3	169.22 (9)	C5—C4—H4A	119.2
O1—Cu1—N5	169.26 (8)	C6—C5—C4	120.3 (3)
N4—Cu1—N5	82.50 (9)	C6—C5—H5A	119.9
N3—Cu1—N5	104.10 (9)	C4—C5—H5A	119.9
O1—Cu1—O3	100.15 (8)	C5—C6—C7	119.3 (3)
N4—Cu1—O3	94.09 (8)	C5—C6—H6	120.3
N3—Cu1—O3	94.71 (8)	C7—C6—H6	120.3
N5—Cu1—O3	87.45 (8)	C8—C7—C6	117.2 (2)
C3—N1—N2	110.7 (2)	C8—C7—C14	118.2 (3)
C3—N1—H1	124.6	C6—C7—C14	124.5 (3)
N2—N1—H1	124.6	N4—C8—C7	123.0 (2)
C2—N2—N1	102.5 (2)	N4—C8—C9	116.3 (2)
C3—N3—C2	103.2 (2)	C7—C8—C9	120.6 (2)
C3—N3—Cu1	148.06 (19)	N5—C9—C10	123.3 (2)
C2—N3—Cu1	108.37 (16)	N5—C9—C8	116.8 (2)
C4—N4—C8	118.4 (2)	C10—C9—C8	119.9 (2)
C4—N4—Cu1	128.81 (18)	C9—C10—C11	117.4 (3)
C8—N4—Cu1	112.74 (17)	C9—C10—C15	118.6 (3)
C13—N5—C9	117.7 (2)	C11—C10—C15	124.0 (3)
C13—N5—Cu1	130.77 (19)	C12—C11—C10	118.8 (3)
C9—N5—Cu1	111.51 (17)	C12—C11—H11	120.6
O5—N6—O4	121.3 (3)	C10—C11—H11	120.6
O5—N6—O3	119.4 (3)	C11—C12—C13	120.3 (3)
O4—N6—O3	119.3 (2)	C11—C12—H12	119.8
C1—O1—Cu1	116.28 (17)	C13—C12—H12	119.8
N6—O3—Cu1	125.19 (17)	N5—C13—C12	122.4 (3)
O2—C1—O1	125.5 (2)	N5—C13—H13	118.8
O2—C1—C2	121.4 (2)	C12—C13—H13	118.8
O1—C1—C2	113.0 (2)	C15—C14—C7	121.5 (3)
N2—C2—N3	114.1 (2)	C15—C14—H14	119.3
N2—C2—C1	128.3 (2)	C7—C14—H14	119.3
N3—C2—C1	117.6 (2)	C14—C15—C10	121.2 (3)
N1—C3—N3	109.4 (2)	C14—C15—H15	119.4
N1—C3—H3	125.3	C10—C15—H15	119.4
N3—C3—H3	125.3		
			
C3—N1—N2—C2	0.2 (3)	O1—C1—C2—N2	174.8 (3)
O1—Cu1—N3—C3	−178.1 (4)	O2—C1—C2—N3	−178.1 (3)
N4—Cu1—N3—C3	−133.1 (5)	O1—C1—C2—N3	−1.0 (4)
N5—Cu1—N3—C3	−6.3 (4)	N2—N1—C3—N3	−0.2 (3)
O3—Cu1—N3—C3	82.3 (4)	C2—N3—C3—N1	0.2 (3)
O1—Cu1—N3—C2	10.49 (17)	Cu1—N3—C3—N1	−171.5 (3)
N4—Cu1—N3—C2	55.4 (5)	C8—N4—C4—C5	−0.7 (4)
N5—Cu1—N3—C2	−177.73 (17)	Cu1—N4—C4—C5	−178.4 (2)
O3—Cu1—N3—C2	−89.18 (18)	N4—C4—C5—C6	1.1 (4)
O1—Cu1—N4—C4	−5.8 (2)	C4—C5—C6—C7	0.1 (4)
N3—Cu1—N4—C4	−50.4 (6)	C5—C6—C7—C8	−1.7 (4)
N5—Cu1—N4—C4	−178.8 (2)	C5—C6—C7—C14	−179.9 (3)
O3—Cu1—N4—C4	94.3 (2)	C4—N4—C8—C7	−0.9 (4)
O1—Cu1—N4—C8	176.37 (18)	Cu1—N4—C8—C7	177.13 (19)
N3—Cu1—N4—C8	131.8 (4)	C4—N4—C8—C9	178.6 (2)
N5—Cu1—N4—C8	3.38 (17)	Cu1—N4—C8—C9	−3.4 (3)
O3—Cu1—N4—C8	−83.50 (18)	C6—C7—C8—N4	2.1 (4)
O1—Cu1—N5—C13	139.0 (4)	C14—C7—C8—N4	−179.5 (2)
N4—Cu1—N5—C13	179.9 (3)	C6—C7—C8—C9	−177.4 (2)
N3—Cu1—N5—C13	8.6 (3)	C14—C7—C8—C9	1.0 (4)
O3—Cu1—N5—C13	−85.6 (3)	C13—N5—C9—C10	−0.9 (4)
O1—Cu1—N5—C9	−43.7 (5)	Cu1—N5—C9—C10	−178.6 (2)
N4—Cu1—N5—C9	−2.82 (17)	C13—N5—C9—C8	179.5 (2)
N3—Cu1—N5—C9	−174.14 (17)	Cu1—N5—C9—C8	1.8 (3)
O3—Cu1—N5—C9	91.65 (18)	N4—C8—C9—N5	1.0 (3)
N4—Cu1—O1—C1	175.3 (2)	C7—C8—C9—N5	−179.5 (2)
N3—Cu1—O1—C1	−12.3 (2)	N4—C8—C9—C10	−178.6 (2)
N5—Cu1—O1—C1	−144.3 (4)	C7—C8—C9—C10	0.9 (4)
O3—Cu1—O1—C1	81.2 (2)	N5—C9—C10—C11	−1.6 (4)
O5—N6—O3—Cu1	−148.4 (2)	C8—C9—C10—C11	178.0 (2)
O4—N6—O3—Cu1	32.6 (3)	N5—C9—C10—C15	178.1 (2)
O1—Cu1—O3—N6	−52.7 (2)	C8—C9—C10—C15	−2.3 (4)
N4—Cu1—O3—N6	−142.7 (2)	C9—C10—C11—C12	2.6 (4)
N3—Cu1—O3—N6	31.0 (2)	C15—C10—C11—C12	−177.0 (3)
N5—Cu1—O3—N6	135.0 (2)	C10—C11—C12—C13	−1.3 (5)
Cu1—O1—C1—O2	−172.5 (2)	C9—N5—C13—C12	2.4 (4)
Cu1—O1—C1—C2	10.6 (3)	Cu1—N5—C13—C12	179.5 (2)
N1—N2—C2—N3	−0.1 (3)	C11—C12—C13—N5	−1.3 (5)
N1—N2—C2—C1	−176.0 (3)	C8—C7—C14—C15	−1.6 (4)
C3—N3—C2—N2	0.0 (3)	C6—C7—C14—C15	176.7 (3)
Cu1—N3—C2—N2	175.33 (18)	C7—C14—C15—C10	0.2 (4)
C3—N3—C2—C1	176.3 (2)	C9—C10—C15—C14	1.8 (4)
Cu1—N3—C2—C1	−8.3 (3)	C11—C10—C15—C14	−178.5 (3)
O2—C1—C2—N2	−2.3 (5)		

### Hydrogen-bond geometry (Å, °)

**Table:**

*D*—H···*A*	*D*—H	H···*A*	*D*···*A*	*D*—H···*A*
N1—H1···O2^i^	0.86	1.92	2.775 (3)	172

Symmetry codes: (i) −*x*+1, *y*+1/2, −*z*+3/2.
